# The involvement of McpB chemoreceptor from *Pseudomonas aeruginosa* PAO1 in virulence

**DOI:** 10.1038/s41598-019-49697-7

**Published:** 2019-09-11

**Authors:** Cristina García-Fontana, Juan I. Vílchez, Marta González-Requena, Jesús González-López, Tino Krell, Miguel A. Matilla, Maximino Manzanera

**Affiliations:** 1 Institute for Water Research and Department of Microbiology, University of Granada, Granada, Spain; 2 Department of Environmental Protection, Estación Experimental del Zaidín, Consejo Superior de Investigaciones Científicas, Prof. Albareda 1, 18008 Granada, Spain

**Keywords:** Environmental microbiology, Soil microbiology, Biotechnology

## Abstract

*Pseudomonas aeruginosa* is an opportunistic human pathogen causing infections in a variety of plant and animal hosts. The gene *mcpB*, part of the chemosensory gene cluster II, encodes a soluble chemoreceptor whose function remains unknown. Previous studies show that the *cheB2* gene, also located in the chemosensory cluster II, is involved in a specific response during infection and it is required for full pathogenicity of *P*. *aeruginosa*. To determine whether the McpB (or Aer2) chemoreceptor is involved in virulence processes, we generated a *mcpB* mutant and tested its phenotype using a virulence-measuring system. This system was developed by our group and is based on different bioassays using organisms living at different soil trophic levels, including microbial, nematode, arthropod, annelid, and plant model systems. The deletion of *mcpB* resulted in an attenuation of bacterial virulence in different infection models, and wild-type virulence was restored following genetic complementation of the mutant strain. Our study indicates that the McpB chemoreceptor is linked to virulence processes and may constitute the basis for the development of alternative strategies against this pathogen.

## Introduction

The Gram-negative bacterium *Pseudomonas aeruginosa* is a widespread opportunistic human pathogen responsible for multiple hospital-acquired infections, mainly in immunocompromised and cystic fibrosis patients^1,2^. In addition, the host spectrum of *P*. *aeruginosa* is not restricted to humans since it was also shown to infect different animals and plants^3–8^.

**Figure 1:**
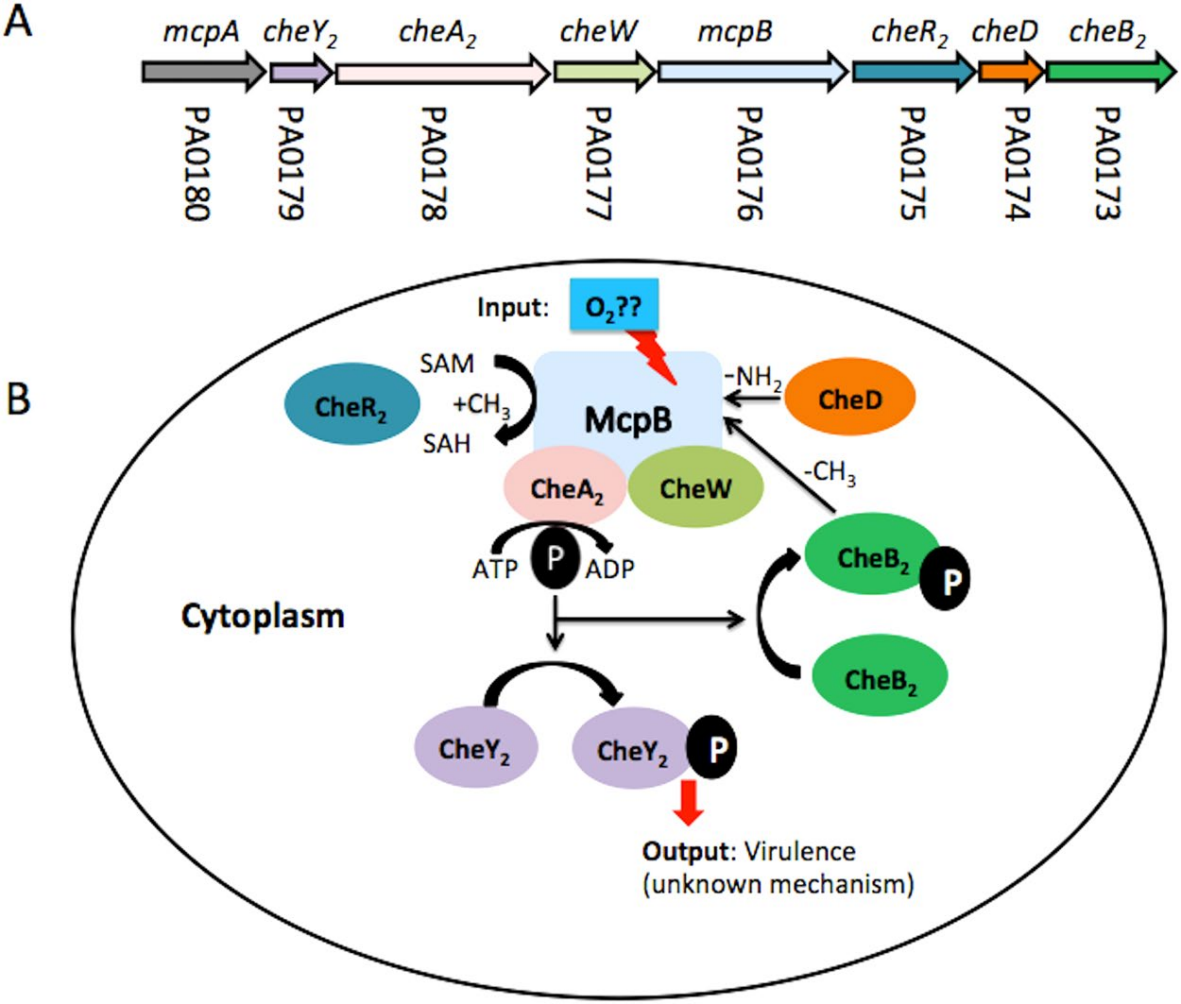
Proposed model of the Che2 signaling pathway. Panel A shows the genes that form part of the gene cluster II encoding the signaling protein of the Che2 pathway. Panel B shows the hypothetical mechanism of the Che2 pathway.”P” phosphoryl group, SAM: S-adenosylmethionine, SAH: S-adenosylhomocysteine. Note: although the *mcpA* gene form part of gene cluster II multiple pieces of evidence suggests that this chemoreceptor may feed into the Che pathway^24^.

To adapt efficiently to environmental changes, *P*. *aeruginosa* has evolved sophisticated regulatory networks that include one- and two-component systems as well as chemosensory signalling pathways^9^. The action of chemosensory pathways is initiated by sensing signal molecules by chemoreceptors^10^ (Fig. 1A). The molecular stimulus resulting from chemoreceptor activation is transmitted to the histidine kinase CheA homologue. The sensitivity of a chemosensory pathway is adjusted by chemoreceptor methylation or demethylation catalysed by the CheR methyltransferase and CheB methylesterase homologues, respectively. The signal output of chemosensory pathways is alterations in the level of phosphorylated CheY homologue (CheY-P). Most chemosensory pathways appear to be involved in chemotaxis^11^, in those cases CheY-P binds to the flagellar motor altering its activity. Other chemosensory pathways are associated with type IV pili-mediated taxis or have been shown to regulate alternative cell functions, such as the modulation of the intracellular levels of the bacterial second messengers cAMP and c-di-GMP^11–13^. Although chemotaxis chemoreceptors having been studied extensively, information on chemoreceptors carrying out alternative functions is scarce.

Proteins encoding chemosensory signalling proteins in *P*. *aeruginosa* PAO1 are encoded by five different gene clusters and form four different chemosensory pathways^9,14^. Gene cluster II encodes proteins of the Che2 pathway, which was originally associated with aerotaxis^15^ but its function remains controversial, since this role in aerotaxis could not be reproduced in subsequent studies^14,16^.

Cluster II consists of eight genes encoding the chemosensory proteins CheB2, CheD, CheR2, CheW2, CheA2 and CheY, as well as the chemoreceptors McpB (Aer2) and McpA^14^ (Fig. 1B). Proteins encoded by cluster II have been shown to form complexes that co-localize at cell poles, and the requirement of McpB for the formation of these complexes has been demonstrated^16^. The McpB lacks transmembrane domains and it has been shown to be a soluble chemoreceptor^17^. The McpB contains a C-terminal pentapeptide (GWEEF) that enables the specific binding of CheR2 and chemoreceptor methylation^17^. The heterologous expression of McpB in a chemoreceptor-deficient strain of *Escherichia coli* showed this chemoreceptor to be responsible for mediating repellent chemotactic responses to oxygen, nitric oxide, and carbon monoxide^18^. The PAS-type ligand binding domain of McpB contains bound heme^18^ and a recent study showed that oxygen is the native ligand of McpB^19^.

Chemotaxis has been shown to play an important role in the pathogenicity of a broad range of bacterial pathogens^20^ and several reports have correlated chemotaxis with virulence of *P*. *aeruginosa*^4,21–23^. One of these studies has linked the Che2 pathway of *P*. *aeruginosa* PA14 with pathogenicity because a mutant defective in *cheB2* showed attenuated virulence in *Caenorhabditis elegans* and murine lung-infection models^4^. A recent bioinformatics study suggests that McpB is the sole chemoreceptor feeding into the Che2 pathway^24^. The notion that the Che2 pathway is involved in virulence stems from high-throughput screening experiments of bacterial mutants that has resulted in the identification of the *cheB2* mutant as the most severely affected mutant; a finding that was verified by experimentation in mice and complementation studies^4^. Very little is known on the Che2 pathway nor on the specificity of the function of the four CheB paralogues of *P*. *aeruginosa*. No information is available on the nature of the pathway output, nor have the receptor(s) that feed into this pathway been identified experimentally.

In the present study we have used multiple bioassays to evaluate the involvement of McpB in bacterial virulence. These assays were based on a diverse range of model organisms recently described to determine the virulence levels of bacterial strains^25^. Our results show that McpB plays a critical role in the virulence and pathogenesis of *P*. *aeruginosa* PAO1 in a variety of hosts.

## Results and Discussion

### *P*. *aeruginosa* PAO1 Δ*mcpB* shows attenuated virulence in *C*. *elegans*

**Figure 2:**
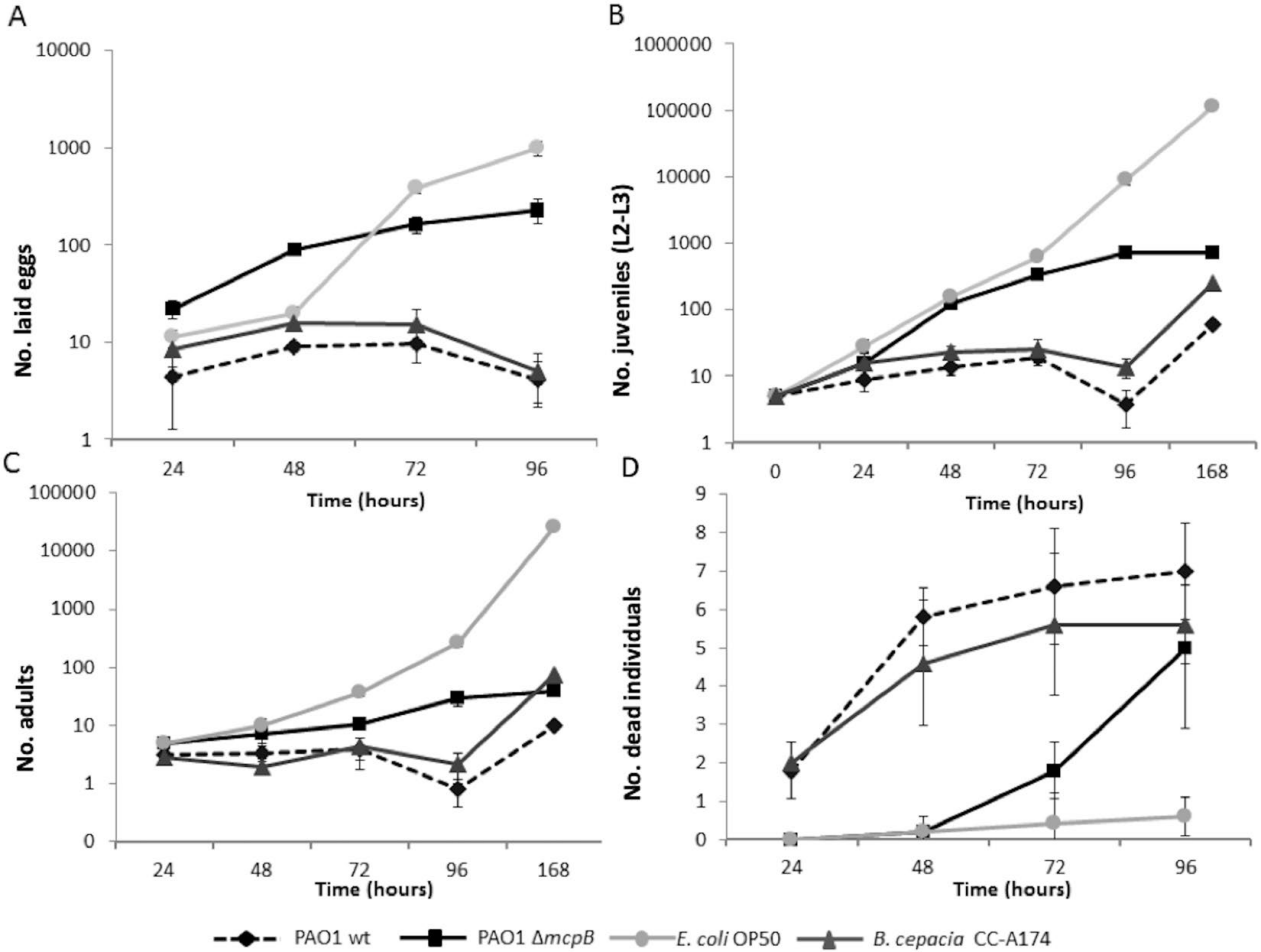
Virulence of *P*. *aeruginosa* strains against *C*. *elegans*. The number of eggs laid (**A**), juveniles at the larval stage L2-L3 (**B**), adults (**C**) and dead *C*. *elegans* individuals (**D**) are shown when cultured with *P*. *aeruginosa* strains. *B*. *cepacia* CC-A174 and *E*. *coli* OP50 were used as pathogenic and non-pathogenic control strains, respectively. Means and standard deviations of three biological replicates are shown.

Virulence factors responsible for the killing of *C*. *elegans* have also been found relevant for virulence in mammalian hosts^26,27^. Previous studies have shown that *P*. *aeruginosa* is virulent in a *C*. *elegans* model^4,28,29^, a model commonly used to study bacterial virulence mechanisms^29–31^. To test the role of McpB in the virulence properties of *P*. *aeruginosa* PAO1, we constructed an in-frame deletion mutant *mcpB*-deficient (PAO1 Δ*mcpB*). Subsequently, we evaluated the effects of the wild-type *P*. *aeruginosa* PAO1 (PAO1 wt) and Δ*mcpB* on the number of eggs laid, number of juvenile and adult nematodes, and death rates^25,32^. Our results showed that the Δ*mcpB* exhibited lower nematicidal activities compared to the PAO1 wt strain (Fig. 2). The number of eggs laid by the nematodes in the presence of the Δ*mcpB* strain resulted in an approximately 10- and 60-fold increase after 24 h and 96 h, respectively, compared to the parental strain (PAO1 wt) using ANOVA (*P* ≤ 0.05; Fig. 2A). Furthermore, the number of juvenile and adult nematodes was significantly higher when PAO1 Δ*mcpB* was used to feed *C*. *elegans* compared to the PAO1 wt strain. Therefore, an increase of approximately 30- and 100-fold in the number of adult and juvenile nematodes, respectively, was observed when PAO1 Δ*mcpB* was used as the feeding source after 96 h compared to the those fed with the PAO1 wt strain (*P* ≤ 0.05, Fig. 2B,C). Additionally, the number of dead nematodes throughout the experiment was significantly lower (*P* ≤ 0.05) when worms were fed on saturated cultures of the Δ*mcpB* strain grown on potato dextrose agar (PDA) plates after 24 h than when they were fed with the PAO1 wt strain (Fig. 2D).

Then, we detected a 4- and 10-fold increase in the survival of the adult and juvenile nematodes, respectively, when they were fed with PAO1 Δ*mcpB* compared to those fed with the PAO1 wt strain (last sampling time, Fig. 2B,C). The virulence properties of *P*. *aeruginosa* PAO1 was similar to that observed in *Burkholderia cepacia* CC-A174, a strain with nematicidal properties used as an internal control.

### Wild-type virulence is restored in PAO1 Δ*mcpB* by *in trans* expression of *mcpB*

**Figure 3:**
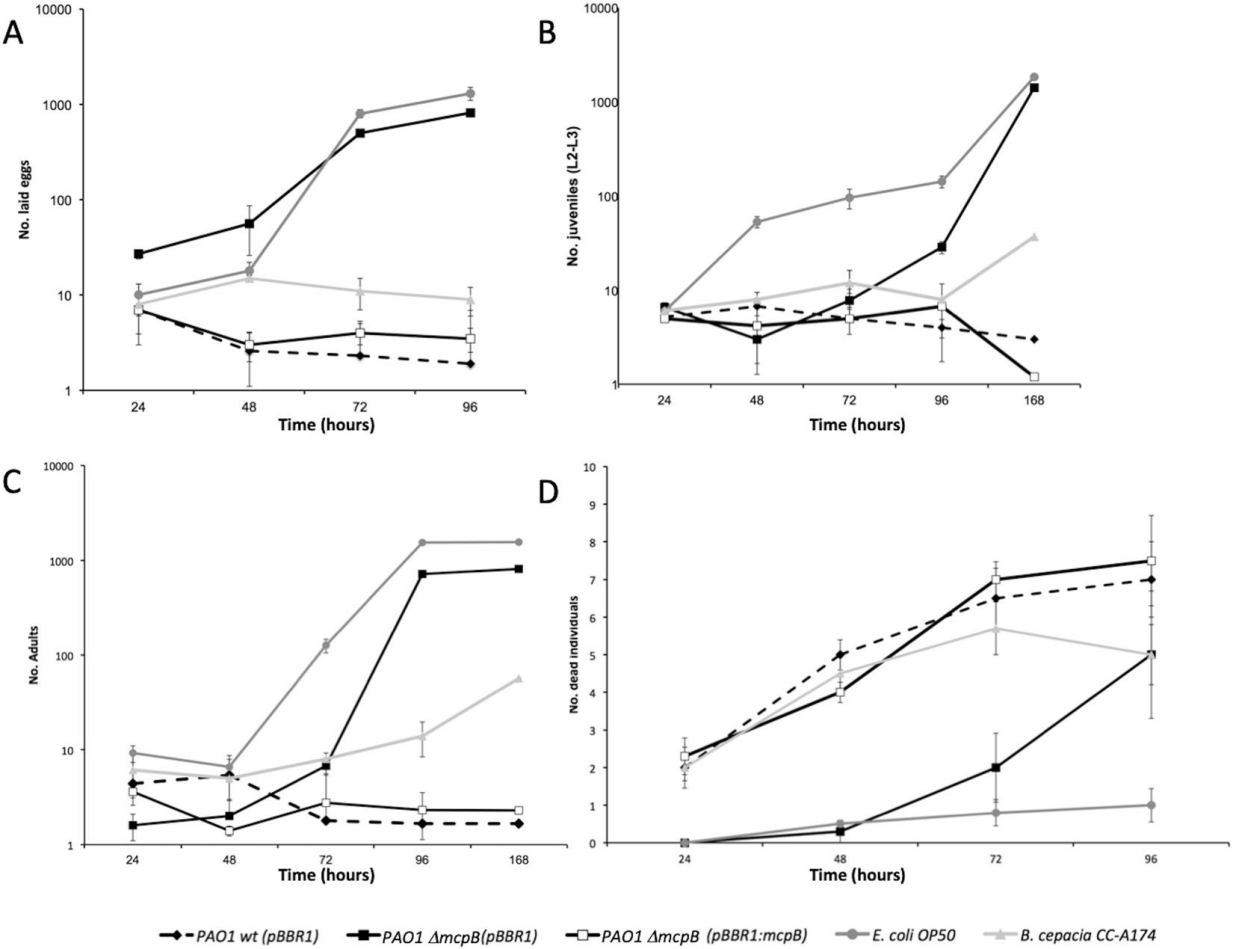
*In trans* expression of *mcpB* restores *P*. *aeruginosa* PAO1 Δ*mcpB* virulence in *C*. *elegans*. Shown are the number of eggs laid (**A**), juveniles (**B**), adults (**C**), and dead individuals (**D**). Both, *P*. *aeruginosa* wt strains PAO1 (solid diamonds and back dotted line) and *P*. *aeruginosa* PAO1 Δ*mcpB* (solid squares and black line) harbour pBBR1MCS-2 (named as pBBR1). McpB complemented *P*. *aeruginosa* PAO1 Δ*mcpB* (white squares and black line) harbours pBBR1:mcpB plasmid. *E*. *coli* OP50 (circles and dark grey lines) was used as non-pathogenic control and *B*. *cepacia* CCA-174 (triangles and light grey lines) was used as pathogenic control. Means and standard deviations of three biological replicates are shown. For statistic analysis ANOVA (*P* ≤ 0.05) was used for comparison of datasets obtained at the end of each experiment.

To confirm the role of McpB in the observed phenotypes, the mutation in *mcpB* was functionally complemented by the expression of the mutated gene *in trans* using the broad host range pBBR1MCS-based plasmid pBBR1:mcpB. Subsequently, the above reported experiments were repeated with the complemented mutant and compared with PAO1 wt and PAO1 Δ*mcpB* strains bearing the pBBR1MSC-2 backbone plasmid. The results showed that the *in trans* expression of *mcpB* restored wt nematicidal properties in the PAO1 Δ*mcpB* (pBBR1:mcpB) strain using ANOVA (*P* ≤ 0.05, Fig. 3). We analyzed plasmid stability in control experiments showing that the plasmid in all *P*. *aeruginosa* strains was highly stable and independent of the presence of antibiotic (data not shown).

Despite the molecular mechanisms of signalling through the Che2 pathway have not been established, currently available data strongly suggest that oxygen is the natural ligand serving as input^19^. In addition to CheB2, we show here that McpB is involved in virulence. This strongly suggests that McpB feeds into this pathway and that it may be the target for CheB2 action. This interpretation is consistent with Garvis *et al*. (2009)^4^, showing a reduction in virulence of the *cheB2* mutant. These authors have shown that a mutant defective in the methylesterase-encoding gene *cheB2* results in reduced virulence in *C*. *elegans* and murine models. Non-mammalian models have been used to study infections caused by *P*. *aeruginosa*. One such model is based on the interaction and subsequent killing of the bacteria-feeding nematode *C*. *elegans* allowing to identify bacterial virulence factors and host responses to them^28^.

### Role of McpB in the insecticidal properties against *Chrysoperla carnea* and *Adalia bipunctata*

**Figure 4:**
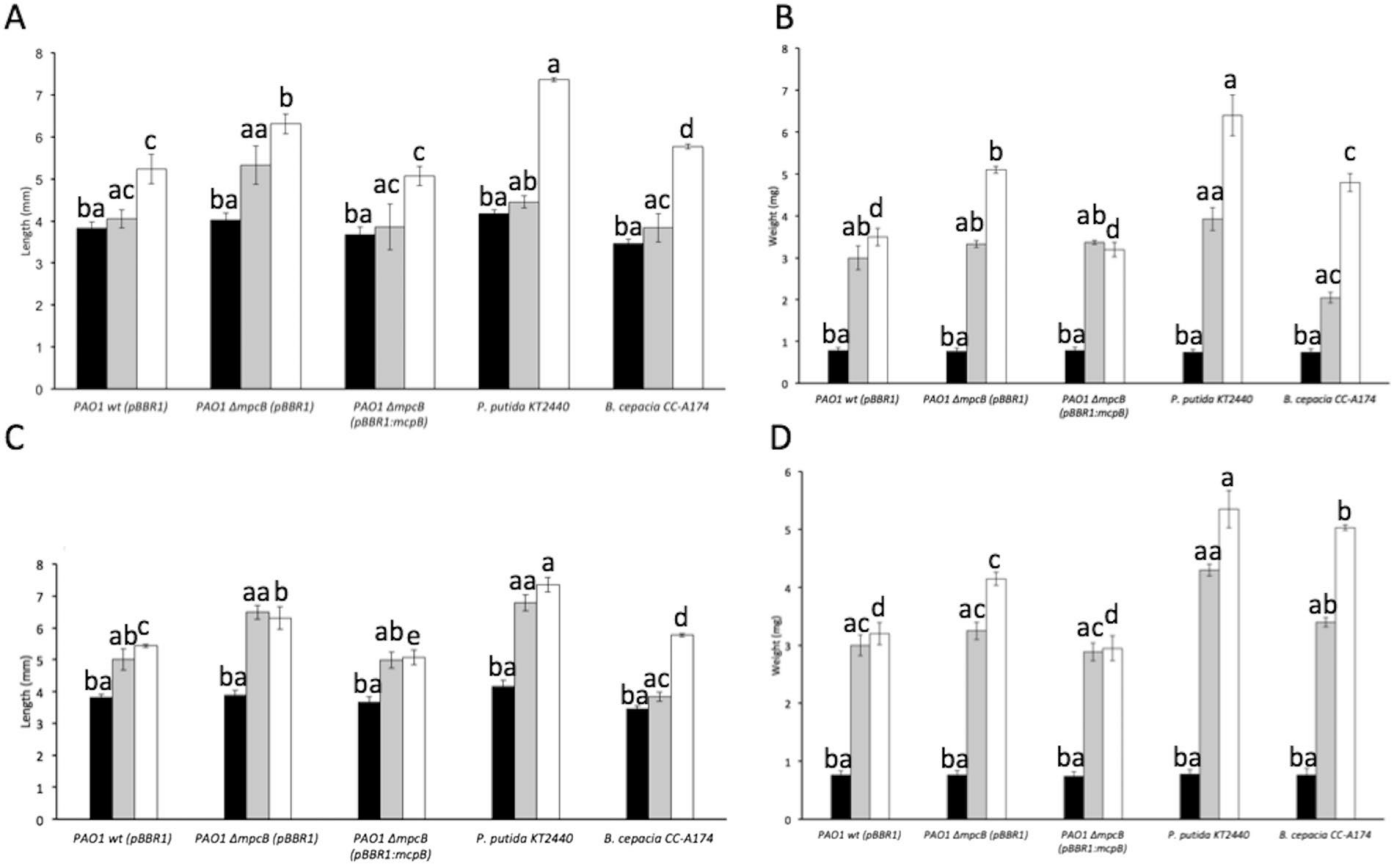
Length and weight of *A*. *bipunctata* and *C*. *carnea* individuals fed with different bacterial strains. Virulence assays against *A*. *bipunctata* (**A**,**B**) and *C*. *carnea* (**C**,**D**) are shown. Measurements were made 0 (dark bars), 7 (grey bars), and 14 days (white bars) after feeding. Means and standard deviations of five biological replicates are shown. Data were submitted to an ANOVA test (posthoc Tukey) and significant differences (*P* ≤ 0.05) are highlighted by different single letters (after 14 days), double letters starting with a (after 7 days), and double letters starting with b (time zero). *B*. *cepacia* and *P*. *putida* KT2440 were used as pathogenic and non-pathogenic bacterial controls, respectively.

*P*. *aeruginosa* is an universal pathogen that, apart from humans, was also shown to infect for example plants, fungi or insects^33^. We used two insect models, green lacewings (*C*. *carnea*) and ladybirds (*A*. *bipunctata*), to test the implication of McpB in the insecticidal properties of *P*. *aeruginosa*. These insects are considered efficient biological control agents and have been previously used to validate the virulence features of different proteobacteria and bacterial pathogens in humans^34,35^. In this test, the bacterial strains were concentrated and supplied in a semidry format mixed with the insect feed. Bacterial virulence was evaluated by scoring the number of dead insects and changes in weight and length after feeding them with PAO1 wt or PAO1 Δ*mcpB* strains using approximately 15 insects per strain or condition. The insecticidal strain *B*. *cepacia* CC-A174 and the non-virulent strain *P*. *putida* KT2440 were included in the assay as controls. Our results have shown that when *C*. *carnea* and *A*. *bipunctata* were fed with *P*. *putida* KT2440, the weight and length of the insects showed similar values to those found when the insects were fed with trehalose (control) in the absence of bacteria. In contrast, the length and weight of the insects was reduced when they were fed with the PAO1 wt. Both, weight and length of the insects were statistically higher (*P* ≤ 0.05) when a PAO1 Δ*mcpB* was used to feed the insects than when PAO1 wt was used, despite not finding differences at time zero among the different treatments. This phenotype was complemented by the expression of the *mcpB* gene *in trans* in the PAO1 Δ*mcpB* (pBBR1:mcpB) (Fig. 4).

**Figure 5:**
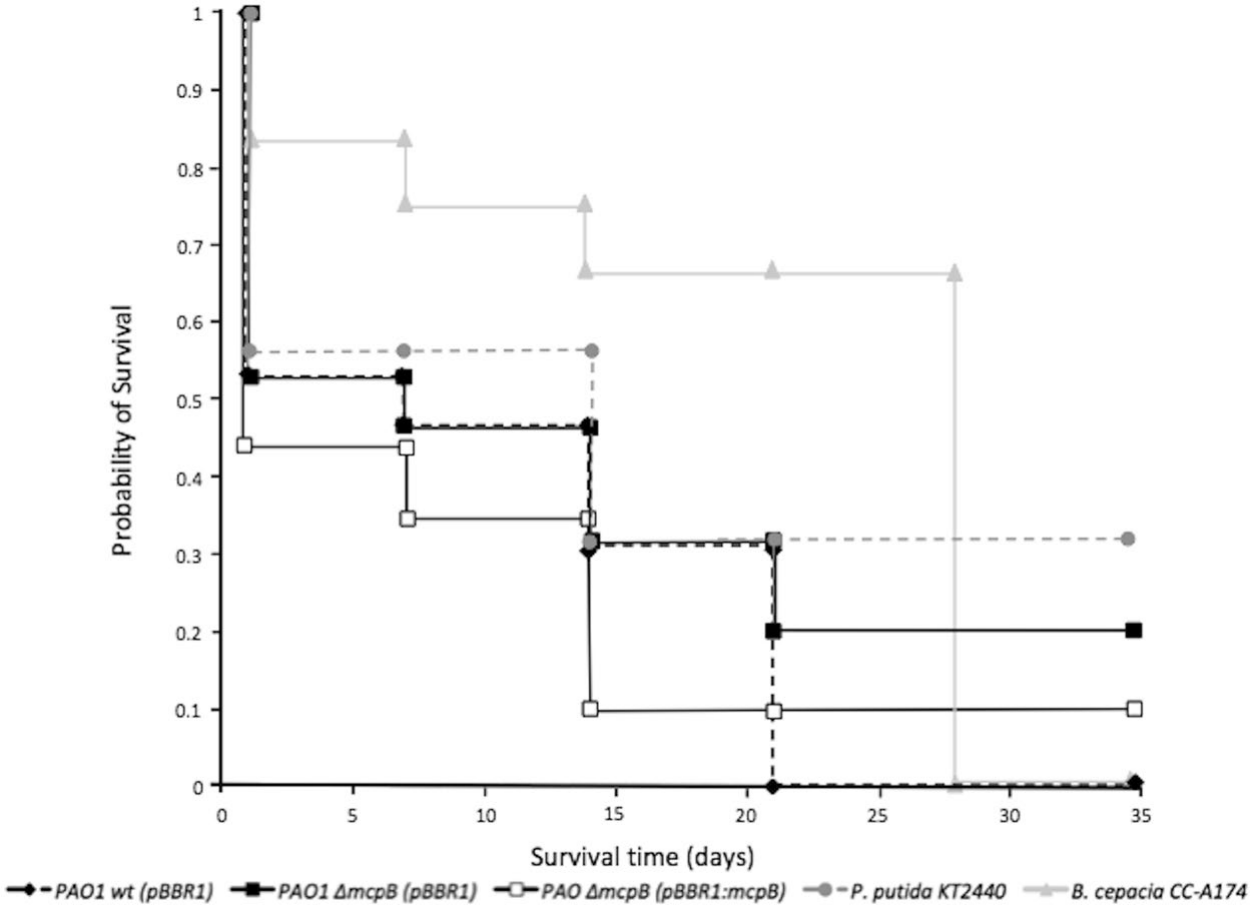
Survival of *C*. *carnea* fed with different bacterial strains. The probability of survival of *C*. *carnea* fed with *P*. *aeruginosa* PAO1 (PAO1 wt (pBBR1)) (solid diamonds and black dotted lines), its *mcpB* mutant PAO1 Δ*mcpB* (pBBR1)) (solid squares and black lines) (both strains carrying the pBBR1MCS-2 plasmid), the complemented *mcpB* strain carrying the pBBR1:mcpB plasmid (PAO1 Δ*mcpB* (pBBR1:mcpB)) (white squares and black lines) as well as for *B*. *cepacia* CC-A174 (pathogenic control) (triangles and light grey lines), *P*. *putida* KT2440 (non-pathogenic control) (circles and dotted dark grey lines) are shown. The figure shows a representative experiment with three biological replicates. For statistic analysis ANOVA (*P* ≤ 0.05) was used for comparison of datasets obtained at the end of each experiment.

The survival of *C*. *carnea* individuals in the presence of *P*. *aeruginosa* PAO1 strains has been also analyzed. PAO1 wt was capable of killing over 80% (12 out of 15) of the individuals after 28 days, whereas PAO1 Δ*mcpB* was severely impaired in virulence and less than 46% of the insects (7 out of 15) were killed after 28 days. When the *mcpB*-restored strain (PAO1 Δ*mcpB* (pBBR1:mcpB)) was used, results were similar to those of the parental strain (13 killed out of 15 insects). This complemented strain showed higher virulence than *B*. *cepacia* for most of the sampling times. The Kaplan-Meier method was used following the description of Bewick *et al*. (2004) to estimate the survival. The comparison of the survival curves of PAO1 wt and PAO1 Δ*mcpB* strains using the log rank tests (Bewick *et al*., 2004) showed significant differences between both strains (Fig. 5). Statistically significant differences between the PAO1 wt (pBBR1) and PAO1 Δ*mcpB* (pBBR1:mcpB) and PAO1 Δ*mcpB* strains has been also observed when experiments were conducted using *A*. *bipunctata* as the model organism (Fig. S1). Burrowes *et al*. (2006) have reported that the post-transcriptional regulator RsmA exerts control over *cheB2*, since its expression was found to be reduced 10-fold in a *rsmA* mutant background^36^. RsmA has been reported to act in conjunction with small non-coding RNA to regulate the expression of multiple virulence genes, including the quorum-sensing *lasI* and *rhlI* genes^37^. The authors proposed that the Che2 signalling pathway may be regulated by RsmA, which constitutes further support to the suggestion that cluster II is involved in virulence^36^. Biofilm tests performed showed that the *mcpB* mutant strain did not alter the biofilm production despite of finding similar number of CFUs for all different strains (data not shown). The concentrated format for the supply of bacterial cells for these insect tests could in theory facilitate a quorum-sensing response, therefore we postulate that the involvement of McpB in virulence occurs in a different manner than the production of biofilms and do not discard a quorum-sensing depending response.

### The deletion of *mcpB* gene causes a reduction in the ecotoxicity towards earthworms

*Eisenia foetida* earthworms present a highly developed immune system^38^. These worms have developed efficient defence mechanisms against microbes that they either ingest or enter their bodies after injury^39–41^. However, several soil-related bacterial pathogens were found to affect the developmental and reproductive capacity of *E*. *foetida*^42^. Since *E*. *foetida* lives underground we could simulate environments of different gradients of oxygen limitations.

**Figure 6:**
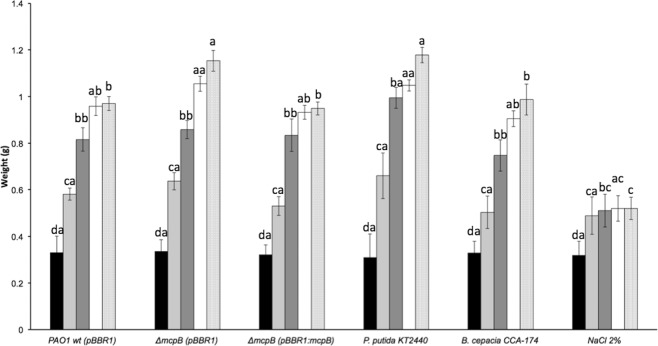
Weight of *E*. *foetida* fed with different bacterial strains or in the presence of trehalose or NaCl. The weight of *E*. *foetida* individuals at 0 (black bars), 7 (light grey bars), 14 (dark grey bars), 21 (white bars), and 28 days (dotted bars) after feeding is shown. *B*. *cepacia* and NaCl 2% (w/v) were used as pathogenic and toxic controls, respectively. *P*. *putida* KT2440 was used as non-pathogenic control. The means and standard deviations of 5 individuals from three biological replicates are shown. Data were submitted to an ANOVA test and significant differences (*P* ≤ 0.05) and posthoc Tukey are highlighted by different single letters (after 28 days), double letters starting with a (after 21 days), double letters starting with b (after 14 days), double letters starting with c (after 7 days) and double letters starting with d (at time zero).

To study the effect of *P*. *aeruginosa* strains on *E*. *foetida* development, we measured weight gain, length gain and the reproductive efficiency (number of juvenile worms and oothecas) of *E*. *foetida* after adding different freeze-dried bacterial strains in trehalose to their diet. Our results showed no difference in the length increase between the earthworms exposed to PAO1 wt and those exposed to PAO1 Δ*mcpB* (data not shown). However, the annelids gained more weight when they were fed with PAO1 Δ*mcpB* compared to the earthworms exposed to PAO1 wt. Furthermore, a similar weight gain was observed when these worms were fed with the *mcpB*-restored strain PAO1 Δ*mcpB* (pBBR1:mcpB), compared to the PAO1 wt (pBBR1), despite not finding differences at time zero among the different treatments (Fig. 6).

The number of eggs laid, and therefore the reproductive efficiency of *E*. *foetida*, significantly increased in the presence of PAO1 Δ*mcpB* compared to the number of eggs laid by PAO1 wt strain and PAO1 Δ*mcpB* (pBBR1:mcpB) strain despite not finding differences at time 7 days among the different bacterial treatments (supplementary Fig. S2A). In addition, the exposure of annelids to the PAO1 Δ*mcpB* strain resulted in more than 4-fold increase in the number of juveniles compared to PAO1 wt (ANOVA *P* ≤ 0.05) at 21 and 28 days (supplementary Fig. S2B), although the same number of juveniles were used at time zero for all different treatments.

A mutant in the methylesterase-encoding gene *cheB1*, which is part of the cluster I, showed no effect on virulence but chemotaxis and motility were significantly attenuated^4^. Altogether, these results suggest that the Che pathway plays the dominant role in *P*. *aeruginosa* chemotaxis and flagellar motility, whereas cluster II is associated with a chemosensory pathway activated during infection due to the sensing of an unknown signal^4^, most probably oxygen^19^. In fact, several studies have shown a link between oxygen levels and virulence of *P*. *aeruginosa*^43,44^. Bioinformatics studies have predicted that a single chemoreceptor, McpB, feeds into the Che2 pathway^24^, which is consistent with studies showing that the deletion of the *mcpB* and *cheB2* genes reduce bacterial virulence^4^. These results together with previous studies conducted by different groups, suggest that the Che2 pathway plays a role in *P*. *aeruginosa* virulence through oxygen sensing^19^. Accordingly, studies performed on different bacteria have established a correlation between oxygen sensing and virulence^20^. For example, the plant pathogen *Ralstonia solanacearum* presents two aerotaxis receptors, termed Aer1 and Aer2. Mutant strains defective in *aer2* or *aer1*/*aer2* have a delayed development of wilt disease in tomato plants upon infection with the *R*. *solanacearum* mutant strains^45^.

### Effect of metabolites secreted by *P*. *aeruginosa* strains on bacterial communities, and crustaceans

Bioactive metabolites produced and released into the environment can alter the composition of microbial communities and their interactions^46–48^. In the particular case of *P*. *aeruginosa* as an animal pathogen, these metabolites can affect host microbiomes, which may result in the development of diseases associated with microbiome alterations^49^. Accordingly, we first evaluated the effect of filter-sterilized supernatants of *P*. *aeruginosa* strains on the viability of *E*. *coli* MC4100, the bacterial metabolism using Microtox^**®**^, and on *Daphnia magna* as a representative of the aquatic ecosystems using DaphToxKit^**®**^. The bioassays showed no statistically significant differences in *E*. *coli* MC4100 survival, in bacterial metabolism using Microtox^**®**^, nor on *D*. *magna* survival using DaphToxKit^**®**^, when these organisms were exposed to the supernatants of PAO1 Δ*mcpB* (pBBR1) strain compared to the supernatants of PAO1 wt (data not shown).

Taken together, our results indicate that the deletion of *mcpB* did not cause any measurable changes in the pool of toxic bioactive metabolites of *P*. *aeruginosa* PAO1.

### *P*. *aeruginosa* PAO1 Δ*mcpB* strain does not affect pepper plants

Some studies describe virulence pathways in *P*. *aeruginosa* that are required for the infection of human and plant hosts^50^. For this reason, virulence assays using pepper plants (*Capsicum annuum*) were conducted. However, no statistical differences were found regardless the strain used (data not shown).

In our study we have used models based on the exposure to bacterial supernatants (such as the DaphToxKit^**®**^, Microtox^**®**^, or the test of viability of *E*. *coli* MC4100) and models based on ingestion of the strain (i.e. *C*. *carnea*, or *A*. *bipunctata*). Interestingly, changes were only observed when the strain was supplied as a component of the organisms’ diet, whereas *mcpB* deletion had no effect when assays evaluated the production of bioactive molecules or toxins secreted to the extracellular medium. This implies that Che2-mediated virulence requires the direct contact between the strain and host cells. Further research is needed to elucidate the output of the Che2 pathway and to understand the molecular mechanism underlying the role of McpB in virulence.

### The deletion of *mcpB* increases the environmental and human safety index (EHSI) of *P*. *aeruginosa*

We have previously proposed the use of the EHSI, an index that combines a set of mortality, reproduction, and development tests, as a value indicative of the virulence potential of a bacterial strain^25^. This index ranges from 0 to 100, with higher values representing higher safety of the bacterial strain^25^. Therefore, the EHSI was used to quantify the differences between the pathogenic capabilities of PAO1 wt (pBBR1) and PAO1 Δ*mcpB* (pBBR1) (Table 1).

**Table 1:** Summary of results obtained with different animal models for the wild-type (PAO1 wt (pBBR1)), *mcpB* mutant (Δ*mcpB* (pBBR1)) and complemented mutant (Δ*mcpB* (pBBR1:mcpB)) strains of *P*. *aeruginosa* PAO1.

Bioassay	PAO1 wt (pBBR1)	Δ*mcpB* (pBBR1)	Δ*mcpB* (pBBR1:mcpB)
EHSI			
*E*. *coli* MC4100	5.00	5.00	5.00
Microtox^®^ (*V*. *fischeri*)	1.25	1.25	1.25
*C*. *elegans*	0	6.00	0
*C*. *carnea*	3.18	6.75	2.18
*A*. *bipunctata*	4.12	6.75	4.12
*E*. *foetida*	8.75	10.62	8.75
*D*. *magna*	1.87	1.87	1.87
*C*. *annuum*	4.00	4.00	4.00
**Final score**	**28.17**	**42.24**	**27.17**

Values were obtained using standard values proposed by Vilchez *et al*. (2016).

These data were used to calculate the Environmental and Human Safety Index (EHSI) following the protocol reported by^25^.

Although both PAO1 strains, wt and Δ*mcpB*, showed an EHSI indicative of pathogenicity, the PAO1 Δ*mcpB* showed a higher safety score than the PAO1 wt strain. This result indicates an attenuation in the bacterial virulence in the PAO1 Δ*mcpB* strain compared to the PAO1 wt strain. This analysis also revealed that complementation of the mutant strain using the pBBR1:mcpB plasmid reduced this index, indicative of a reduction in safety (Table 1). In this context, we established a link between the chemosensory cluster II of *P*. *aeruginosa* PAO1 and the virulence of the strain by using a panel of virulence assays employing different model organisms which can be used to calculate the EHSI^25^. In addition to the *C*. *elegans* and the pepper plant tests we have also included an assay based on *E*. *foetida*. The strong immune system of the earthworm allows us to discriminate between larger effects due to different pathogenic mechanisms. Furthermore, we have also included more sensitive systems, such as DaphToxKit^**®**^, due to the higher sensitivity of *D*. *magna* to toxins and virulence factors.

## Conclusions

We have designed a method to numerically quantify the relevance of potential virulent factors involved in the process of pathogenesis of *P*. *aeruginosa* PAO1 based on a panel of different bioassays. The index called Environmental and Human Safety Index (EHSI) derived from PAO1 wt increased from 28 up to 42 when the *mcpB* gene was in-frame deleted, while the *in trans* complementation of the mutation reduced it back down to 27 showing that the role of McpB in pathogenesis is not specific to a given model but was observed in species as diverse as *C*. *elegans*, *E*. *foetida* or the insects *A*. *bipunctata* and *C*. *carnae*. The action of McpB requires the ingestion of the pathogen since exposure to bacterial supernatants had no effect on *D*. *magna*, *E*. *coli* or *V*. *fisheri*. The simplicity of this approach can be easily extended to test other proteins or genes involved in pathogenesis for comparison of the virulence relevance of each molecule and determine the most effective targets at fighting a relevant pathogen as *P*. *aeruginosa* PAO1, as well as to other pathogens.

## Materials and Methods

### Organisms, culture media, and growth conditions of bacteria

Organisms used in this study are listed in Table 2. Bacterial strains used in the bioassays were routinely grown on tryptic soy broth (TSB medium: 17 g tryptone L^−1^, 3 g phytone L^−1^, 5 g NaCl L^−1^, 2.5 g K_2_HPO_4_ L^−1^, and 2.5 g glucose L^−1^), except for nematode bioassays where potato dextrose medium (PDA: 4 g potato extract L^−1^, 20 g dextrose L^−1^ and 15 g agar powder L^−1^) was used. The growth temperature was 30 °C, except for *P*. *aeruginosa* PAO1 and its variants, which were cultured at 37 °C. For mutant construction, *P*. *aeruginosa* was grown on Luria-Bertani medium (5 g yeast extract L^−1^, 10 g bactotryptone L^−1^ and 5 g NaCl L^−1^) and in basal M9 medium supplied with 1 mM MgSO_4_, 6 mg L^−1^ Fe-citrate, as previously described^51^. For plasmid stability assays, the bacterial strains were plated on tryptone soy agar medium (TSA: 15 g agar L^−1^, 15 g casein peptone (pancreatic) L^−1^, 5 g NaCl L^−1^ and 5 g soy peptone (papainic) L^−1^). When appropriate, antibiotics were used at the following final concentrations (in µg mL^−1^): ampicillin, 100; kanamycin, 25 (*E*. *coli* strains) and 100 (*P*. *aeruginosa* strains); streptomycin, 50 (*E*. *coli* strains) and 400 (*P*. *aeruginosa* strains). Sucrose was added to a final concentration of 10% (w/v) when required to select derivatives that had undergone a second crossover event during marker-exchange mutagenesis.

**Table 2:** Organisms used in this study.

Bacteria/Nematodes/Arthropods/Annelids	Genotype, relevant characteristics and uses^a^	Reference or source
*E*. *coli* MC4100	F- *araDI39* Δ(*argF*-*lac*) *U169 rpsL150 relAl flb5301 deoCI ptsF24 rbsR*. Used to evaluate the presence of antibiotic compounds in supernatants of PAO1 strains.	^56^
*E*. *coli* OP50	Uracil auxotroph. Used to feed *C*. *elegans*.	^57^
E. coli β2163	Km^R^Em^R^; F^−^ RP4-2-Tc::Mu Δ*dapA*::(*erm*-*pir*).	^58^
E. coli DH5α	*supE44 lacU169* (*Ø80lacZΔ M15*) *hsdR17* (rk-mk-) *recA1 endA1 gyrA96 thi*-*1 relA1*.	^59^
*B*. *cepacia* CC-Al74	Wild type. Risk Group 2 bacteria and proposed as plant growth promoting rhizobacteria (PGPR). Used as a pathogenic control in virulence assays.	^60^
*P*. *putida* KT2440	Wild type; Risk Group 1 bacteria and PGPR. Used as negative control in virulence assays.	^61^
*V*. *fischeri* ATCC 49387	Wild type; Used as bioluminescent strain in MicroTox^®^ assays.	^62^
*P. aeruginosa* PAO1	Wild type pathogenic strain.	^9^
*P. aeruginosa ΔmcpB*	In-frame deletion mutant in *mcpB* (1116 bp Δ).	This study
*C*. *elegans* Bristol strain N2	Nematode used in virulence assays. Provided by the Laboratory of Nematology, National Museum of Nature Sciences-CSIC (Madrid, Spain)	Navas *et al*., 2007
*A. bipunctata*	Arthropod used in virulence assays. Supplied by ControlBio Co. (Almería, Spain).	Ref. CBi K04884
*C. carnea*	Arthropod used in virulence assays. Supplied by ControlBio Co. (Almería, Spain).	Ref. CBi 124 K04280
*E. foetida*	Annelid used in virulence assays. Supplied by Lombriventa (Gerona, Spain).	^63^
*D. magna*	Crustacean used in virulence assays. Supplied by Daphtoxkit™ (Creasel, Belgium).	^64^

### Construction of in-frame deletion mutant of PAO1 Δ*mcpB*

Oligonucleotides and plasmids used in this study are listed in Tables S1, S2, respectively. A mutant defective in *mcpB* (PA0176) was constructed using homologous recombination. A derivative plasmid of the suicide vector pKNG101 was used for this purpose. The plasmid for the construction of the in-frame deletion mutant was constructed by amplifying the upstream and downstream flanking regions of *mcpB*. The resulting PCR products were digested with EcoRI and BamHI or BamHI and HindIII for the upstream and downstream regions of *mcpB*, respectively. These products underwent a three-way ligation into pUC18Not in order to generate plasmid pUC18:Δ*mcpB*, previously to be cloned into the NotI site of the marker exchange vector pKNG101. The sequence cloned into the resulting plasmid, called pKNG:Δ*mcpB*, was confirmed by DNA sequencing and it carried an in-frame deletion of *mcpB* gene for the replacement of wt gene in the chromosome. For the construction of the Δ*mcpB* mutant strain, biparental conjugations were performed as previously described^52^. Briefly, in a biparental mating, 100 µl of overnight cultures of *P*. *aeruginosa* PAO1 and *E*. *coli* β2163 harbouring pKNG:Δ*mcpB* were mixed, collected by centrifugation, resuspended in 30 µl of fresh Luria Broth (LB), and spotted on an LB agar plate supplemented with 300 µM 2,6-diaminopimelic acid (DAPA). After overnight incubation at 37 °C, cells were scraped off the plate and resuspended in 1 mL of LB. Serial dilutions were plated on LB agar medium containing 400 µg mL^−1^ streptomycin. DAPA was not added to the LB agar medium plates to avoid *E*. *coli* donor growth. We added sucrose to a final concentration of 10% (w/v) to select derivatives that had undergone a second crossover event during marker-exchange mutagenesis. Final mutants lacking the *mcpB* gene were confirmed using PCR and sequencing.

### Plasmid construction for genetic complementation assays

For the construction of the complementing plasmid, a full copy of the *mcpB* gene was amplified by PCR using the primers mcpB-compl-NdeI-F and mcpB-compl-EcoRI-R listed in Table S1. The resulting fragment was digested with NdeI and EcoRI and cloned into the same sites in pBBR1MCS2_START^53^ to generate pBBR1:*mcpB*. The insert was confirmed by PCR and sequencing, and pBBR1:*mcpB* was used to transform the *mcpB* defective mutant by electroporation.

### Stability assays of plasmids pBBR1 and pBBR1:mcpB in immobilized *P*. *aeruginosa* PAO1 strains

To perform bacterial virulence assays using *C*. *elegans*, the stability of the pBBR1 and pBBR1:mcpB plasmids in *P*. *aeruginosa* PAO1 strains were tested in the absence of antibiotic. Briefly, *P*. *aeruginosa* PAO1 strains were cultured overnight in TSB medium supplemented with kanamycin (50 µg mL^−1^) at 37 °C in an orbital shaker at 200 r.p.m. Subsequently, 200 µl from each culture medium were spread on PDA plates and incubated at 37 °C for 5 h until a thin growth film was visible. Plates were kept at 20 °C for one week to reproduce *C*. *elegans* biosafety test conditions. Biomass from each strain was resuspended in 1 mL of M9 salts medium and serial dilutions were plated onto TSA plates in the presence or absence of kanamycin (50 μg mL^−1^). Plasmid stability was determined by calculating the ratio between CFU mL^−1^ in the presence or absence of antibiotic.

### Biofilm assays and quantification

Biofilm formation was quantified using a borosilicate glass tubes (hydrophilic surface) following the methodology described by O’Toole *et al*. with some modifications^54^. Briefly, the borosilicate glass tubes were inoculated with 2 mL aliquots of LB cultures of *P*. *aeruginosa* WT and Δ*mcpB* at A_600_ of 0.05 in triplicate. The tubes were incubated at 37 °C at 40 rpm for 5 h. Unattached cells were removed by rinsing the tubes thoroughly with water. Attached cells were subsequently stained with 0.3% crystal violet and incubated at 37 °C for 15 min. Then, tubes were washed twice with water to remove any unbound stain. Crystal violet was then solubilized with 5 mL of 10% (v/v) glacial acetic acid and spectrophotometrically quantified at 590 nm. Biofilm formation was normalized with the corresponding cell density.

### Virulence assays

Virulence and ecotoxicity of *P*. *aeruginosa* strains were evaluated using a combination of bioassays that were performed as described previously by our group^25^. These assays included antibacterial activity against *E*. *coli* MC4100, microbial metabolism assays using *Vibrio fischeri* (Microtox^**®**^; Modern Water), pathogenicity bioassay against *Caenorhabditis elegans*, ecotoxicity tests using green lacewings (*Chrysoperla carnea*), ladybirds (*Adalia bipunctata*), earthworms (*Eisenia foetida*) and *Daphnia magna* (DaphToxKit®; Microbiotest), and bacterial effects on pepper (*Capsicum annuum* L. cv. Maor) plants. In each case, bioactivities were compared with those of non-pathogenic and pathogenic bacterial strains, or with trehalose as carrier of the dry formats as previously described^25^. The number of replicates is stated in the description of each experiment.

### Bacterial effects in pepper (*Capsicum annuum*) plants

Virulence in pepper plants was tested according to Vílchez *et al*.^25^ with some modifications. Pots containing pepper plants of about 10 cm height were inoculated with a 4 mL of bacterial suspension (10^8^-10^9^ CFU/mL) in 0.5 X M9 sterile saline solution. Plants were irrigated twice a week with 4 mL water per plant. Fourteen days after inoculation, height, fresh weight, fully turgid weight and dry weight were recorded. As a control, pepper plants were amended with saline buffer.

### Statistical analyses

The analysis of variance (ANOVA) test was used to determine the effects of treatments and errors associated with the experiment with replicates and treatments as random effects. The Statistical Analysis Software (SAS) version 9.1 was used (SAS Institute Inc., Cary, NC). Means were compared to identify significant differences among treatments. The protected least significance difference (LSD) (*P* ≤ 0.05) test was used for this purpose; mean square error obtained in this test was used to estimate the standard error of differences between means. The survival function S(t) was calculated as the probability of surviving at least to time t to analyse the survival data with *C*. *carnea* and *A*. *bipunctata*. The survival curve was represented using S(t) against t. The Kaplan-Meier method was used to generate survival curves from the observed survival times without the assumption of an underlying probability distribution. This analysis was based on the assumption that the probability of surviving k or more periods from entering the study is a product of the k observed survival rates for each period, following the formula:

$${\rm{S}}({\rm{k}})={{\rm{p}}}_{1}\times {{\rm{p}}}_{2}\times {{\rm{p}}}_{3}\times \,\ldots \,\times {{\rm{p}}}_{{\rm{k}}}$$

where p_1_ is the proportion surviving the first period, p_2_ is the proportion surviving the second period, and so on. Therefore, for a specific period i the proportion of survival was calculated as:

$${\rm{pi}}=({{\rm{r}}}_{{\rm{i}}}-{{\rm{d}}}_{{\rm{i}}})/{{\rm{r}}}_{{\rm{i}}}$$

where r_i_ is the number of alive insects at the beginning of the period and d_i_ the number of dead insects within the period^55^. Comparison of the survival curves obtained with different bacterial strains was performed using the log rank test as statistical hypothesis.

## Supplementary information

The involvement of McpB chemoreceptor from Pseudomonas aeruginosa PAO1 in virulence
