# Supplementary material for: The involvement of McpB chemoreceptor from Pseudomonas aeruginosa PAO1 in virulence
Source: Sci Rep. 2019 Sep 11;9:13166. doi: 10.1038/s41598-019-49697-7 (PMC6739360; doi:10.1038/s41598-019-49697-7)
Supplement: Supplementary file 1 — The involvement of McpB chemoreceptor from Pseudomonas aeruginosa PAO1 in virulence [file 41598_2019_49697_MOESM1_ESM.docx]

**SUPPLEMENTARY INFORMATION**

**The involvement of McpB chemoreceptor from *Pseudomonas aeruginosa* PAO1 in virulence**

Cristina García-Fontana^1^, Juan I. Vílchez^1^, Marta Gonzalez-Requena^1^, Jesús González-López^1^, Tino Krell^2^, Miguel A. Matilla^2^ and Maximino Manzanera^1*^

1. Institute for Water Research and Department of Microbiology, University of Granada, Granada, Spain.
2. Department of Environmental Protection, Estación Experimental del Zaidín, Consejo Superior de Investigaciones Científicas, Prof. Albareda 1, 18008 Granada, Spain.

* Corresponding author: Maximino Manzanera

Address: Institute for water Research. CL. Ramón y Cajal No. 4. Granada. 18071. Phone: +34 958 248324. Fax: +34 958 243094. E-mail address: manzanera@ugr.es

**Running Title:** McpB chemoreceptor and virulence

**Table S1. Oligonucleotides used in this study.**

| Name | Sequence (5´-3´) | Description | Reference |
| --- | --- | --- | --- |
| mcpB-up-EcoRI-F | TAAT**GAATTC**CACTGAGGCAAGCCAGGCG | Forward primer to clone upstream flanking region of *mcpB* for in-frame deletion | This study |
| mcpB-up-BamHI-R | TAAT**GGATCC**ACCTGCTGGAGTTCGGC | Reverse primer to clone upstream flanking region of *mcpB* for in-frame deletion | This study |
| mcpB-dw-BamHI-F | TAAT**GGATCC**GAATGCCCGCCAGGCCAACTC | Forward primer to clone downstream flanking region of *mcpB* for in-frame deletion | This study |
| mcpB-dw-HindIII-R | TAAT**AAGCTT**TCAGAACTCTTCCCAGCCGTCT | Reverse primer to clone downstream flanking region of *mcpB* for in-frame deletion | This study |
| mcpB-compl-NdeI-F | TAAT**CATATG**GGTCTGTTCAATGCACATGCGG | Forward primer to generate the plasmid for complementation assays | This study |
| mcpB-compl-EcoRI-R | TAAT**GAATTC**GGAGGTGGGCATTGGCGTCG | Reverse primer to generate the plasmid for complementation assays | This study |

**Table S2. Plasmids used in this study.**

| **Plasmids** | **Relevant characteristics and uses^a^** | **Reference** |
| --- | --- | --- |
| pUC18NotI | Ap^R^; identical to pUC18 but with two NotI sites flanking pUC18 polylinker. | Herrero *et al.*, 1990 |
| pKNG101 | Sm^R^; *oriR6K mob sacBR.* | Kaniga *et al.*, 1991 |
| pBBR1MCS-2_START | Km^R^; *oriRK2 mobRK2* | Obranić *et al*., 2013 |
| pUC18:Δ*mcpB* | Ap^R^; 1.4-Kb PCR product containing a 1116 bp in-frame deletion of *mcpB* inserted into the EcoRI/HindIII sites of pUC18Not. | This study |
| pKNG:Δ*mcpB* | Sm^R^; 1.5-kb NotI fragment of pMAMV249 was cloned at the same site in pKNG101. | This study |
| pBBR1:*mcpB* | Km^R^; *mcpB* gene was cloned into NdeI and EcoRI sites of pBBR1MCS-2_START | This study |

^a^Ap, ampicillin; Km, kanamycin; Sm, streptomycin; Em, erythromycin

**
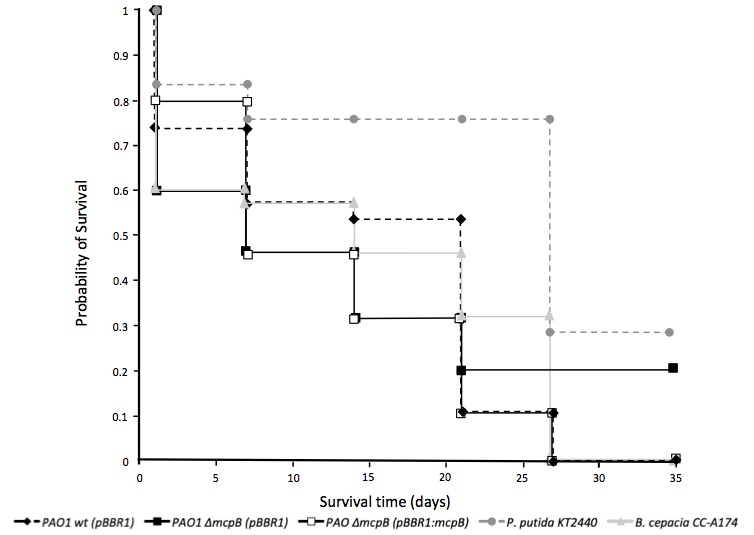
Figure S1. Survival of *A. bipunctata* fed with different bacterial strains.** The probability of survival of two controls, *B. cepacia* CC-A174 (pathogenic control) and *P. putida* KT2440 (non- pathogenic controls), and the survival of the wild-type *P. aeruginosa* PAO1 and its mutant in the *mcpB* gene, both carrying the pBBR1MCS-2 plasmid, as well as the restored *mcpB* strain carrying the pBBR1:mcpB plasmid. A representative experiment with three biological replicates is shown.

**
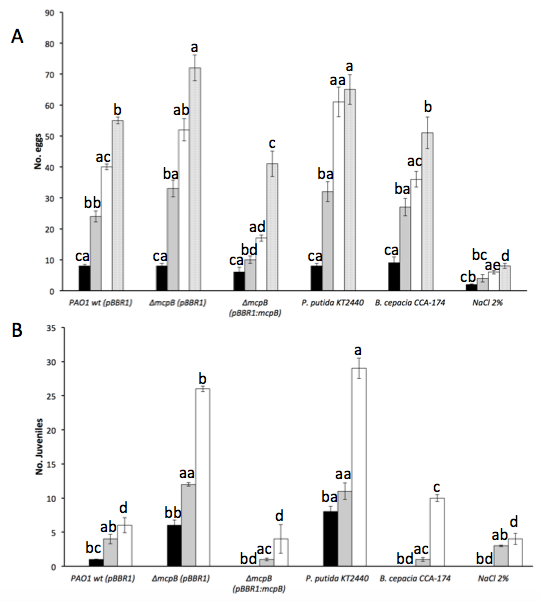
Figure S2. Reproductive efficiency of *E. foetida* fed with different bacterial strains or in the presence of NaCl**. Eggs laid (panel A) and juveniles (panel B) were monitored in 5 individuals at 7 (black bars), 14 (grey bars), 21 (white bars), and 28 days (dotted bars). Means and standard deviations of 5 individuals in three biological replicates are shown. *B. cepacia* and NaCl 2% (w/v) were used as pathogenic and toxic controls, respectively. *P. putida* KT2440 was used as a non-pathogenic control. For panel A, different single letters indicate statistically significant differences among treatments at day 28, double letters starting with a indicate differences at 21 days and double letters starting with b indicate differences at 14 days and double letters starting with c indicate differences at 7 days based on the ANOVA test (*P* ≤ 0.05) and posthoc Tukey. For panel B, different single letters indicate statistically significant differences among treatments at day 21, double letters starting with a indicate differences at 14 days and double letters starting with b indicate differences at 7 days based on the ANOVA test (*P* ≤ 0.05) and posthoc Tukey.
